# Biomechanical comparison of percutaneous posterior endoscopic cervical discectomy and anterior cervical decompression and fusion on the treatment of cervical spondylotic radiculopathy

**DOI:** 10.1186/s13018-019-1113-1

**Published:** 2019-03-04

**Authors:** Jiabin Ren, Rui Li, Kai Zhu, Xuexin Han, Xin Liu, Yu He, Zhaozhong Sun

**Affiliations:** 1 Department of Spinal Surgery, Binzhou Medical University Hospital, No. 661 Huanghe 2nd Road, Binzhou, 256603 Shandong China; 2 Department of Nursing, Binzhou Medical University Hospital, No.661 Huanghe 2nd Road, Binzhou, 256603 Shandong China; 3 Department of Orthopaedics, Peking Union Medical College Hospital, Chinese Academy of Medical Sciences and Peking Union Medical College, No.1 Shuaifuyuan Wangfujing, Beijing, 100010 China

**Keywords:** Posterior endoscopy, Resection of intervertebral disc, Anterior fusion, Cervical spondylosis, Biomechanics

## Abstract

**Background:**

Cervical spondylotic radiculopathy is a common spinal disease. The traditional surgical treatment consists of anterior cervical decompression and fusion (ACDF), but it presents problems such as trauma and fusion complications. Percutaneous posterior endoscopic cervical discectomy (PPECD) is a new minimally invasive technology that has produced good clinical outcome, but further biomechanical comparisons are needed to guide the clinical work. The goal of this study was to compare the biomechanical characteristics of the two methods by finite element analysis.

**Method:**

On the basis of the computed tomography scanning data of five cases of cervical spondylosis after PPECD surgery, five cases after ACDF surgery, and five non-surgical patients, software (Mimics 15.0, HyperMesh 12.0, and Abaqus 6.13) was adopted to establish a C1–C7 segment 3D finite element model. We also applied 50 N vertical load on the C1 surface and 1.5 Nm torque, simulated the anteflexion, rear protraction, and left and right lateral flexion and rotation, and observed the stability, stress distribution, and Cobb angular change of the surgical section of the cervical vertebra under different working conditions.

**Result:**

The postoperative model under different working conditions demonstrated poorer stability than the non-surgical group, but the stability of the PPECD group was close to that of the non-surgical group. The stability of the ACDF group was the worst, especially when making lateral bending and posterior extension. The ACDF group also showed significant differences. The PPECD group showed uniform stress distribution, whereas the ACDF group was under large stress, which was primarily concentrated in the internal fixation system. In addition, the implant showed the potential for fracture. The Cobb angle of surgery section of the PPECD group was smaller than that of the ACDF group, and the stability of the section was good.

**Conclusion:**

From the perspective of finite element analysis, the cervical vertebrae after PPECD treatment showed good biomechanical performance and stability.

## Background

Cervical spondylotic radiculopathy (CSR), which was first described by Ando [1] in 1952, can be treated through non-surgical and surgical approaches. In 1944, Spurling and Scoville [2] first recommended that the posterior intervertebral foramen decompression can safely and effectively treat CSR, but cervical pain and muscle sequelae occur after the operation. The first case of anterior cervical disc resection and fusion surgery was accomplished by Smith and Robinson [3] in 1958, and a good clinical outcome was obtained. Cloward [4] then reported cervical intervertebral fusion using tenon-type implant and introduced neurostructural decompression to treat the cartilaginous endplate under direct vision. From these studies, cervical intervertebral fusion developed to anterior cervical decompression and fusion, and this technology has been demonstrated to be safe and effective [5]. The technology produces a high fusion rate and is regarded as the gold standard for treating CSR due to cervical disc herniation [6]. However, this technique also brings certain problems, such as dysphagia, post-craniotomy haematoma, recurrent laryngeal nerve paralyses, leakage of cerebrospinal fluid, oesophageal perforation, Horner’s syndrome, intervertebral cage displacement, adjacent segment degeneration, pseudoarticulation formation, and other issues brought by fusion [7–10]. With the technological development, many surgical techniques have emerged. Compared with the traditional technologies, novel surgical techniques yield similar surgical outcome while being less damaging to the tissues, exhibit low blood loss, and entail short hospital stay [11–13]. Ruetten et al. [14] first reported posterior cervical intervertebral disc resection by percutaneous endoscopic surgery in 2007. In a random comparison study on the treatment of 175 cases of nerve-root type cervical spondylosis by percutaneous posterior endoscopic cervical discectomy (PPECD) and anterior cervical decompression and fusion (ACDF), with 2 years of follow-up, Ruetten et al. [15] reached a remarkable achievement ratio of 87.4% after PPECD treatment. This result demonstrated that PPECD is a safe and effective replacement of traditional ACDF technology. However, the selection of an appropriate surgical method for clinical CSR treatment remains controversial because an ideal surgical method not only needs to produce obvious verifiable curative effect but should also meet the mechanical stability of postoperative physiological requirements. Many scholars believe that PPECD exerts minimal damage to the anatomical structure and offers good postoperative biomechanical performance [16]. However, studies on the biomechanics of PPECD have been lacking. Therefore, a comparison of postoperative PPECD biomechanics with ACDF after the treatment of nerve-root cervical spondylosis was conducted to compare the biomechanical features of the two procedures.

## Materials and methods

This study was approved by the Ethics Committee of our hospital. All patients provided written informed consent before the initiation of the study.

### Experimental data

Fifteen patients with single-level nerve-root cervical spondylosis treated in our department between October 2017 and January 2018 were selected. These patients (aged 41–62 years; median age 48.5 years) all exhibited lateral root syndrome. Computed tomography (CT) and magnetic resonance imaging examination showed C5/6 lateral protrusion, which correlated with the clinical symptoms and signs. No cervical instability was observed on the dynamic X-ray of the cervical spine. Moreover, no calcification of the herniated disc was found. Among these patients, eight cases were males, and seven cases were females. The three groups were divided based on the different treatment methods: five cases underwent PPECD, five cases underwent ACDF group, and five cases that had non-surgical treatment formed the sCS group.

### Finite element modeling and analysis

Thin-layer spiral CT scanning was conducted on the cervical vertebra of the non-surgical patients, patients with PPECD, and patients with ACDF (GE, USA). CT parameters were as follows: source voltage of 120 kV, current of 100 mA, and layer thickness of 0.625 mm. Before CT scanning, the CT instrument was reset to zero and corrected. A total of 421–625 pieces of the original 2D CT scanning images were obtained and stored in the *.DICOM format.

The obtained 3D models were modeled for the scanning images for the three groups of patients. We entered the original CT data in the DICOM format into the 3D image reconstruction software (Mimics 15.0, Materialise Company, Belgium), established the 3D models, and output to HyperMesh 12.0 (Altair Company, USA) for mesh generation. The material property of various tissues and internal fixators was homogeneous and isotropic, and the specific assignment parameters are shown in Table 1. The number of cells and nodes of the finite element model and various internal fixators are shown in Table 2.

**Table 1:** Material properties of finite element method (FEM) models

Material	Elasticity modulus (MPa)	Poisson’s ratio
Bone tissue		
Cortical bone	18,000	0.3
Cancellous bone	200	0.2
Ligament	Nonlinear spring	
Implant		
Titanium plate	114,000	0.3
Cage	4100	0.4

**Table 2:** Average unit number and node number of finite model

Material	Unit number	Node number
Cervical		
C1	34126	8892
C2	51187	13337
C3	37811	10037
C4	36560	9727
C5	41505	11052
C6	46967	12440
C7	59001	14919
Disc		
C2-C3	2404	885
C3-C4	2655	959
C4-C5	2529	894
C5-C6	3529	1119
C6-C7	4959	1543
Implant		
Titanium plate	18184	5096
Cage	12102	3424

An analysis was conducted using finite element analysis software (Abaqus 6.13; 3DS, Waltham, MA). All the nodes of the end plate under the finite element model C7 were restrained and made its degree of freedom at the directions of *X*, *Y*, and *Z* zero. A 50 N vertical load was applied in the vertical direction of C1, and horizontal torque of 1.5 Nm was applied at the front and back side and the left and right side to simulate the anteflexion, rear protraction, left and right lateral flexion, and left and right rotation, respectively. The range of motion of the cervical vertebra in each direction was calculated and its stability and stress condition was analyzed under such working conditions as vertical load, anteflexion, rear protraction, lateral flexion, and rotation. The local stability of the surgical section was analyzed, and the Cobb angle change of the surgical section was calculated.

### Statistical analysis

Statistical analyses were performed with SPSS 19.0 (SPSS Inc., Chicago, IL) software. The Kolmogorov-Smirnov test was used to assess the normal distribution of the data, and the Bartlett test was used to analyze their homogeneity. Comparisons of the cervical vertebrae activity, overall stability, and Cobb angle under different working conditions were obtained using a separate one-way analysis of variance (ANOVA). The level of statistical significance was defined as *P* < 0.05.

## Results

Based on the effective range previously reported in the literature [17], each of the range of plane motion of the cervical vertebra model confirmed that the modeling method in this study was effective.

### Overall range of motion and stability

**Fig. 1:**
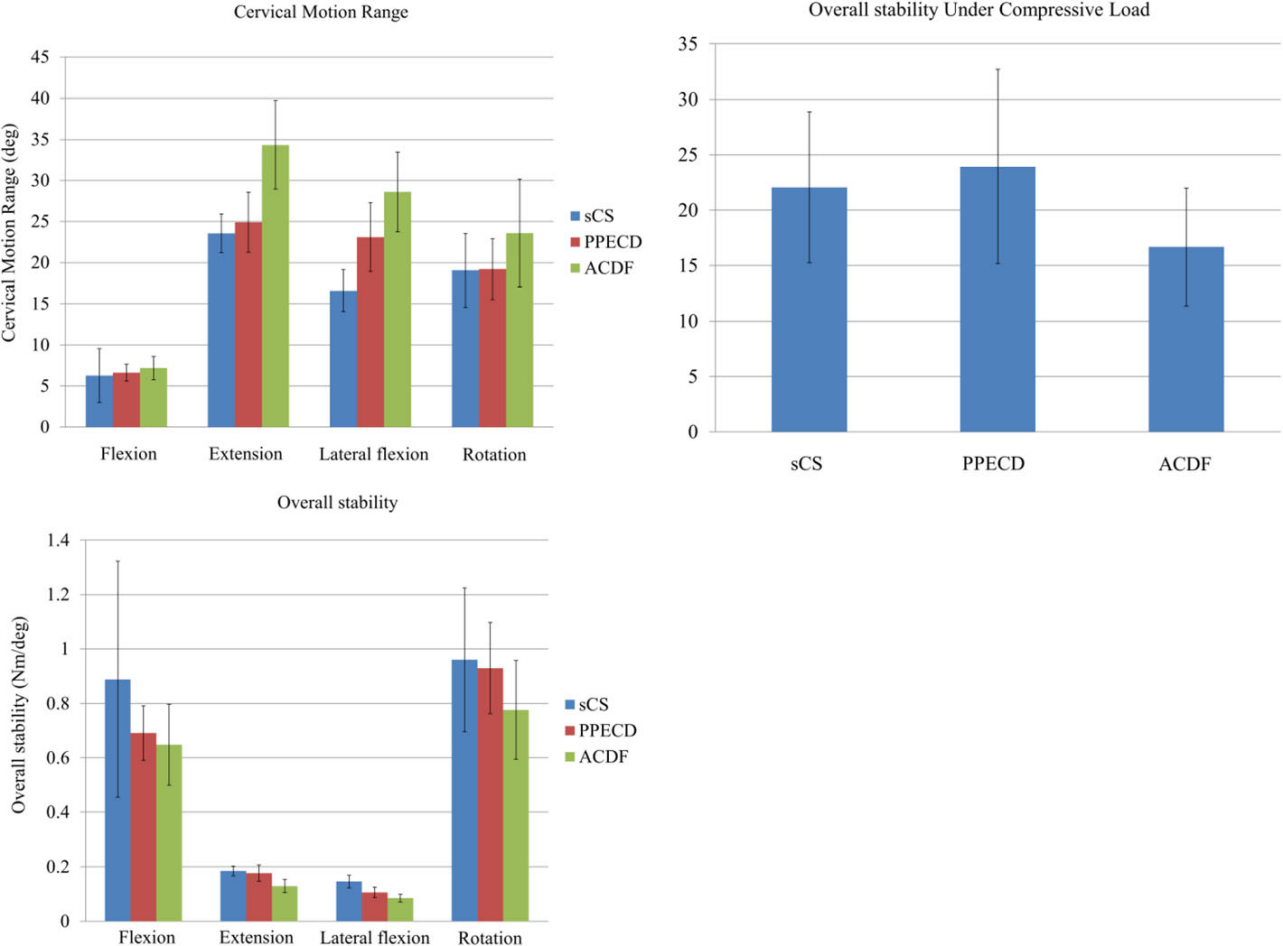
Overall range of motion and stability

The ranges of motion of the non-surgical group when making anteflexion, rear protraction, lateral flexion, and rotation movements were 6.274 ± 3.289°, 23.576 ± 2.358°, 16.596 ± 2.546°, and 19.066 ± 4.502°, respectively. The ranges of motion of the PPECD group under different working conditions were 6.628 ± 1.030°, 24.914 ± 3.652°, 23.114 ± 4.197°, and 19.205 ± 3.684°, respectively. And those of the ACDF group were 7.196 ± 1.419°, 34.336 ± 5.385°, 28.608 ± 4.849°, and 23.601 ± 6.563°, respectively. Under the different working conditions, the non-surgical group had the smallest range of motion, followed by the PPECD group. The ACDF group had the largest range of motion. No significant difference was observed when making anteflexion, rear protraction, and rotation (*P* > 0.05), but the range of motion of the ACDF group statistically differed at 28.608 ± 4.849° (*P* < 0.05) when making lateral flexion (Fig. 1). When the ACDF group achieved the same average range of motion, the minimum applied rear protraction torque of the postoperative cervical vertebra was 0.129 ± 0.024 Nm, showing significant difference (*P* < 0.05). However, under the other stress states, the cervical vertebra of the PPECD and non-surgical groups was more stable than that of the ACDF group, but the difference was not significant.

### Stress distribution

**Fig. 2:**
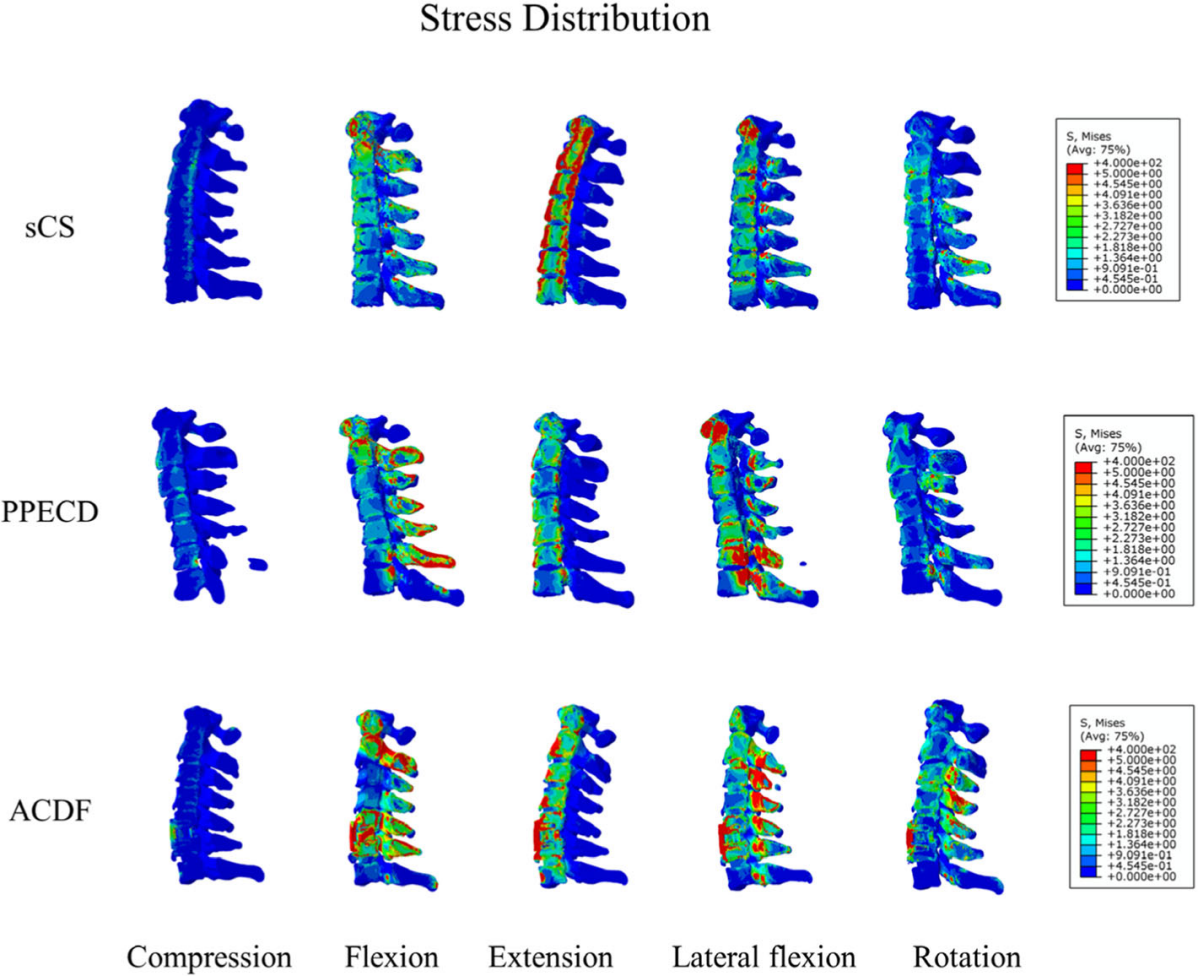
Cervical vertebra overall stress distribution figure

The stresses that the ACDF group bore were concentrated and mainly distributed in the implant of the surgical section. The extreme value of stress under vertical load, anteflexion, rear protraction, lateral flexion, and rotation were 19.6, 631.5, 462.0, 379.9, and 103.2 MPa, respectively. The stress applied in the non-surgical group and the PPECD group scattered through the entire cervical vertebra, and the extreme value of stress was relatively small (Fig. 2 and Table 3).

**Table 3:** Cervical vertebra overall stress extreme value (Mpa)

	Vertical load	Anteflexion	Rear protraction	Lateral flexion	Rotation
sCS	18.22	215.1	227.1	198.8	68.8
PPECD	19.7	537.2	358.5	275.2	76.35
ACDF	19.57	631.5	462	379.9	103.2

### Local biomechanics

**Fig. 3:**
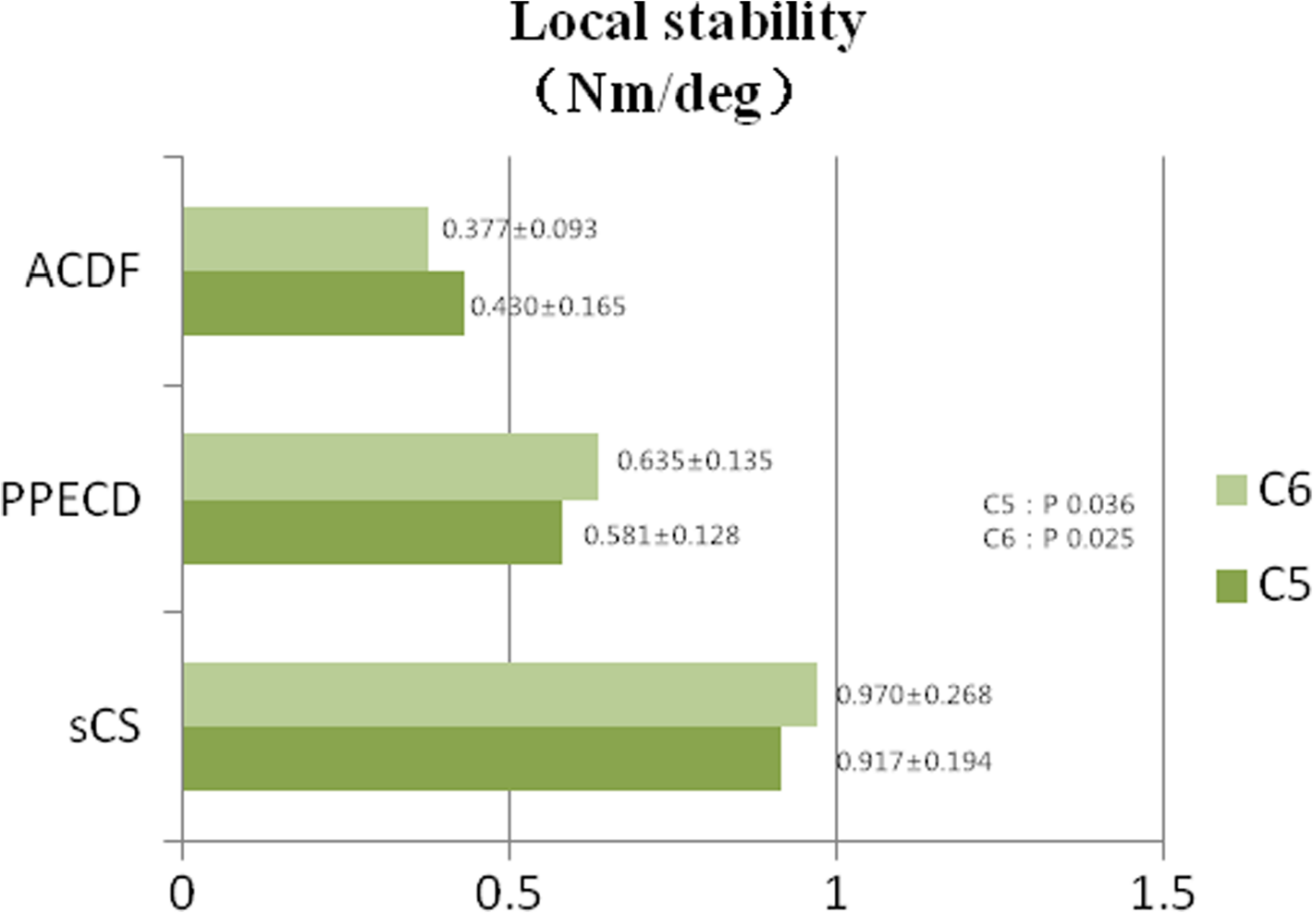
Local stability (Nm/degree)

**Fig. 4:**
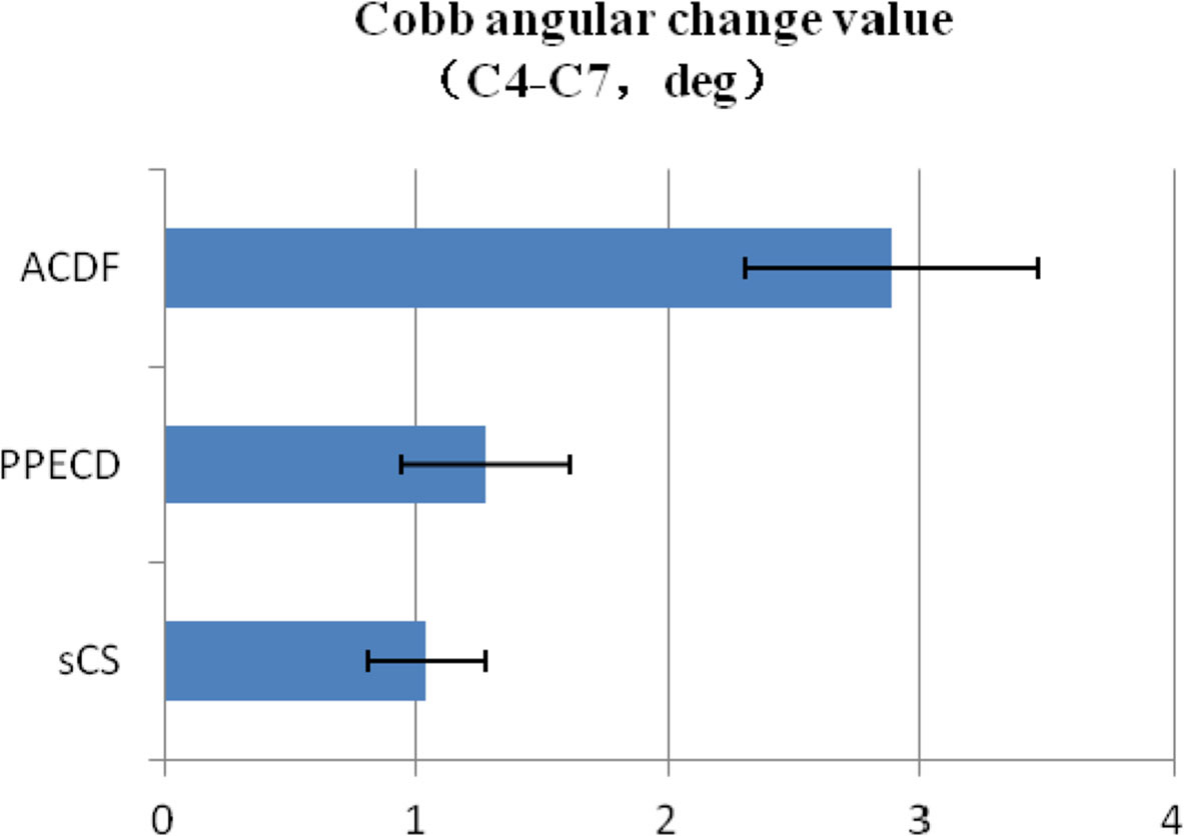
Cobb angular change value (C4–C7, degree)

The C5 and C6 sections of the PPECD group achieved the same average degree of motion, and the required maximum torques were 0.581 and 0.635 Nm, respectively; the stability of the PPECD group was better than that of the ACDF group (Fig. 3). The Cobb angle change of the three groups statistically differed (*P* < 0.05). The Cobb angle change of the non-surgical group was 1.039 ± 0.232°, and that of the PPECD group was 1.272 ± 0.335°. These angles were slightly smaller than that of the ACDF group (2.886 ± 0.577°; Fig. 4).

## Discussion

The ligaments and complex structure that constitute intervertebral disc and facet joints are important structures that maintain the stability of the cervical vertebra [18]. The annulus fibrosus and nucleus pulposus constituting the intervertebral disc consist of different anatomic forms, and biochemical components and yield different biomechanical performance. These structures enable the intervertebral disc to become a unique complexus of solid state and liquid state components and provide sufficient strength and elasticity that can bear the substantial movement of the spine in daily life [19]. In the ACDF group, the intervertebral disc and anterior longitudinal ligament are excised, and the implant did not fuse in the early stage. In our study, the ACDF group presented the worst overall stability, especially when making lateral flexion and rear protraction; the differences were statistically significant. However, limited surgical resection of the vertebral plate or articular process in the PPECD group resulted in maximal retention of the triarticular complex and surrounding ligaments. Under different states of stress, postoperative cervical vertebra demonstrated a similar overall degree of motion and stability to that of the non-surgical group.

During the development of the anterior surgery, it underwent decompression without implantation to decompression with implantation and then to decompression implantation and internal fixation. Internal fixation increased the immediate postoperative local stability and promoted sacralization. Under physiological conditions, the motion was successively transferred from the occipital bone. However, after the ACDF operation, the motion was no longer delivered through interactions of tissues, similar to the entire cervical vertebra. To a large extent, the internal fixation plays a role in delivering motion and bears a great burden. Our study results also verified that the stress that the ACDF group bore was large and mainly concentrated in the internal fixation system, easily producing fractures and implant transposition. In addition, its low fusion rate could result in the formation of pseudarthrosis. However, no rigid implantation was observed, and the stress distribution was homogeneous. The postoperative biomechanical performance also showed minimal changes.

The progressive angular loss of the surgical section after the operation of the cervical vertebra, especially for those patients with less than 10° anterior cervical projection, is a problem that is worth discussing. In terms of the Cobb angle in this study, no significant change was observed after the PPECD operation, whereas that of the ACDF group changed considerably. This finding was consistent with the follow-up study of Kim et al. [16] on the PPECD postoperative sagittal angle imaging of the cervical spine. Our work also verified that the stability of the ACDF operation section was poorer than that of PPECD, which easily caused vertebrae hyperostosis, and calcification of ligamental soft tissue, and probably produced new neurothlipsis.

Our study verified that treatment of CSR by PPECD technology could retain the motor unit of the cervical vertebra and exerted minimal influence on the biomechanical performance of the cervical vertebra. PPECD offers several advantages, such as accurate surgery, low surgical trauma, and rapid recovery. Thus, it has a good application prospect as a novel minimally invasive technology. However, we also need to recognize that this procedure is a treatment intermediate between a conservative treatment and interbody fusion in the CSR ladder treatment. It is necessary to grasp the indications [20] and choose the right patient to achieve the best outcome and minimize the complications.

Given that the spine structure is complex and that motion is multijunctional and multidirectional, with multiple changes in material characteristics, finite element analysis certainly has limitations as a simulation experiment because it cannot reflect the polytrope of the internal individual, external bone, and material characteristics. In addition, this experiment is not a large sample study. Thus, the result obtained from the study only reflected a tendency. Considering that the posterior total endoscopic technique of the cervical spine is an advanced technology with few clinical applications, and that reports on the treatment of multiple segmental cervical disc herniations using this technology are scarce, this study only selected patients with single-level cervical disc herniation. Moreover, whether multiple segment surgery influences the biomechanical stability requires further studies. Further development and completion of the finite element technology are also warranted.

## Conclusions

Although the anterior cervical decompression and fusion is the gold standard for treating CSR, the resection of the posterior cervical vertebral disc by posterior total endoscopic resection of the nucleus pulposus is an alternative technique. 3D finite analysis indicated that the postoperative stability of PPECD was better than that of ACDF. This finding is particularly significant when performing rear protraction and lateral flexion. The postoperative stress of PPECD showed a uniform distribution and had no risk of implant fracture. The operation had minimal influence on the physiological curvature, and the overall performance of biomechanics was good.

## Abbreviations

ACDF: Anterior cervical decompression and fusion
CSR: Cervical spondylotic radiculopathy
CT: Computed tomography
PPECD: Percutaneous posterior endoscopic cervical discectomy
